# Piezoelectric effect in chemical vapour deposition-grown atomic-monolayer triangular molybdenum disulfide piezotronics

**DOI:** 10.1038/ncomms8430

**Published:** 2015-06-25

**Authors:** Junjie Qi, Yann-Wen Lan, Adam Z. Stieg, Jyun-Hong Chen, Yuan-Liang Zhong, Lain-Jong Li, Chii-Dong Chen, Yue Zhang, Kang L. Wang

**Affiliations:** 1 School of Materials Science and Engineering, University of Science and Technology Beijing, Xueyuan Road 30, Beijing 100083, China; 2 Department of Electrical Engineering, University of California, Los Angeles, California 90095, USA; 3 Institute of Physics, Academia Sinica, Taipei 115, Taiwan; 4 California NanoSystems Institute (CNSI), University of California-Los Angeles, Los Angeles, California 90095, USA; 5 WPI Center for Materials Nanoarchitectonics (MANA), National Institute for Materials Science, Tsukuba 305-0044, Japan; 6 Department of Physics and Center for Nanotechnology, Chung Yuan Cristian University, Chungli 32023, Taiwan; 7 Physical Sciences and Engineering Division, King Abdullah University of Science and Technology (KAUST), Huwal 23955-6900, Kingdom of Saudi Arabia

## Abstract

High-performance piezoelectricity in monolayer semiconducting transition metal dichalcogenides is highly desirable for the development of nanosensors, piezotronics and photo-piezotransistors. Here we report the experimental study of the theoretically predicted piezoelectric effect in triangle monolayer MoS_2_ devices under isotropic mechanical deformation. The experimental observation indicates that the conductivity of MoS_2_ devices can be actively modulated by the piezoelectric charge polarization-induced built-in electric field under strain variation. These polarization charges alter the Schottky barrier height on both contacts, resulting in a barrier height increase with increasing compressive strain and decrease with increasing tensile strain. The underlying mechanism of strain-induced in-plane charge polarization is proposed and discussed using energy band diagrams. In addition, a new type of MoS_2_ strain/force sensor built using a monolayer MoS_2_ triangle is also demonstrated. Our results provide evidence for strain-gating monolayer MoS_2_ piezotronics, a promising avenue for achieving augmented functionalities in next-generation electronic and mechanical–electronic nanodevices.

## Abstract (web summary)

Two-dimensional transition-metal-dichalcogenide materials should have strong piezoelectric properties, making them useful for nanosensors and piezotronics. Here, the authors experimentally demonstrate the piezoelectric effect in monolayer molybdenum disulfide and show how this can modulate conductivity.

## Graphical abstract

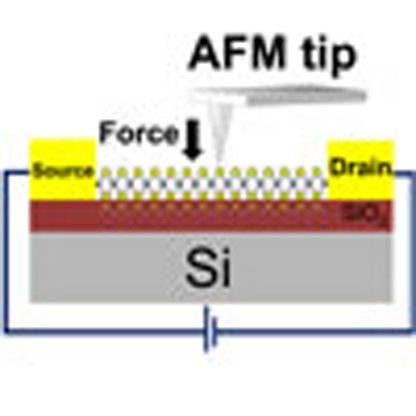

Strain engineering is a powerful strategy for significantly enhancing the performance of electronic and photonic devices^1^,^2^,^3^,^4^,^5^,^6^,^7^. In recent years, many experimental works have used one-dimensional (1D) piezoelectric nanomaterials as the building blocks of piezo-phototronic devices for light emission or as nanogenerators^8^,^9^,^10^. However, inconsistent performance of such devices due to the inhomogeneous as-synthesized 1D materials^11^,^12^,^13^ and difficulties in implementing 1D nanostructure-based devices impede their further applications. Alternatively, utilizing piezoelectric effects in two-dimensional (2D) materials may overcome the limitations caused by 1D nanostructures and could fully take advantage of the state-of-art microfabrication technologies. Considering their ultra-high strain tenability and technological compatibility^14^,^15^,^16^,^17^,^18^,^19^,^20^,^21^,^22^, 2D materials are of great interest as high-performance piezoelectric materials.

Many 2D materials including h-BN, graphene, graphene nitride (C3N4) and trigonal prismatically coordinated transition metal dichalcogenide (TMDC) crystals have demonstrated piezoelectric effects and provide opportunities in the development of novel nano-electromechanical devices^23^,^24^,^25^,^26^. Graphene is not intrinsically piezoelectric but can exhibit piezoelectric effects through introduction of specific defects^25^. Recent reports of piezoelectricity in graphene nitride nanosheets were attributed to the intrinsic existence of nanoscale holes^26^. 2D semiconducting TMDC such as MoS_2_ are gaining increased attention for next-generation electronics and optoelectronics^27^,^28^,^29^,^30^,^31^,^32^ due to their unique properties^33^,^34^,^35^,^36^. Bulk TMDC crystals exhibit a honeycomb structure where adjacent sites occupied by two alternating species are centrosymmetric^37^. Because of this centrosymmetric structure, piezoelectricity is unexpected in bulk TMDC materials. By scaling TMDC thickness down to monolayer, the structure becomes non-centrosymmetric. Owing to the absence of an inversion centre in its crystalline structure, monolayer TMDC is predicted to be strongly piezoelectric^24^. Recently, the piezoelectricity of a mechanically exfoliated MoS_2_ flake on a flexible substrate designed for piezotronics and energy conservation was reported^38^. In their work, polarization charges are induced along one direction at the zigzag edge under uni-axial strain. As compared with exfoliated MoS_2_ flakes, chemical vapour deposition (CVD)-grown monolayer MoS_2_ has a regular triangular shape with three zigzag edges^39^,^40^,^41^. Strain-induced polarization charges are theoretically predicted to accumulate only at zigzag edges under isotropic mechanical deformation^24^ and improved strain-induced piezoelectricity in monolayer MoS_2_ triangles has not been rigorously investigated to date. Therefore, much experimental confirmation about piezoelectricity in CVD-grown monolayer MoS_2_ triangle under mechanical deformations is needed. In addition, although the field of semiconducting TMDC-based electronic devices has evolved rapidly, studies on sensor development have been relatively limited^42^,^43^. In spite of the straightforward concept in piezoelectricity, its potential in electromechanical devices remains largely under exploited.

In the following, we report the experimental study of the piezoelectric effect in CVD-grown monolayer MoS_2_ triangles under isotropic mechanical deformation. The application on a new piezotronic strain/force sensor that is built using a monolayer MoS_2_ with high sensitivity is also presented. The highest gauge factor of the monolayer MoS_2_ strain sensor is ∼1,160, which is much larger than that of conventional metal sensor (1∼5). The working principle of this new type of strain sensor is discussed in comparison with a theoretical model, where polarization charges accumulated at three zigzag edges in the monolayer MoS_2_ triangle enable multi-directional sensor applications. The discovery of this property in 2D materials enables active sensing, actuating and new electronic components for nanoscale devices based on the well-established piezoelectric effect.

## Results

### Characterization of atomic monolayer MoS_2_

**Figure 1:**
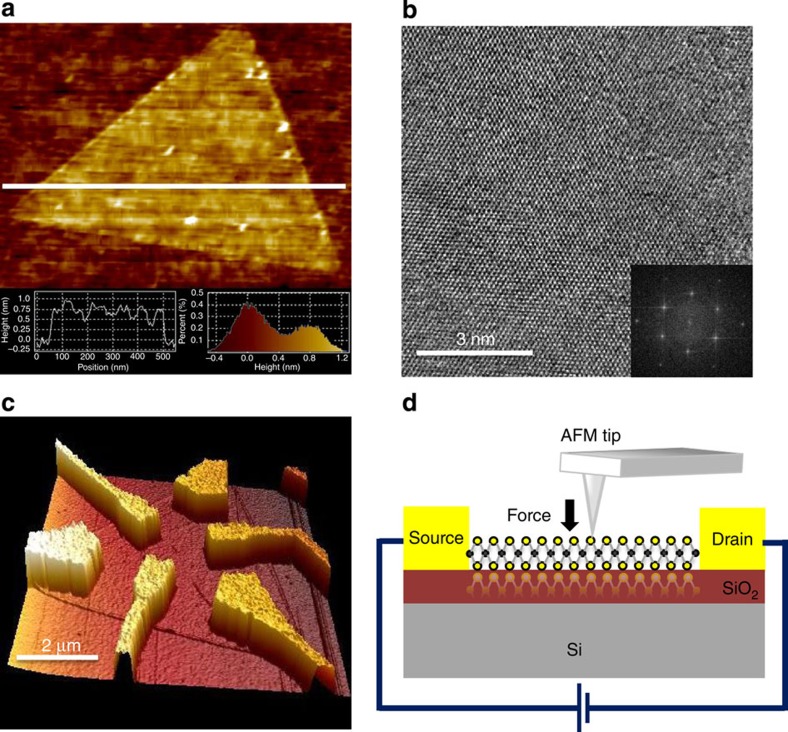
Characterization of the MoS_2_ monolayer and device structure. (**a**) AFM image of a triangular MoS_2_ monolayer. Inset shows the histogram analyses of multiple topographic AFM images confirmed the MoS_2_ film thickness to be ∼0.75 nm. (**b**) High-resolution TEM image of the synthesized MoS_2_ monolayer. Inset is the corresponding diffraction pattern. (**c**) A typical AFM image of a MoS_2_ monolayer device. (**d**) Schematic illustration of a MoS_2_ device under mechanical load applied by an AFM tip.

High-quality monolayer MoS_2_ films were synthesized by CVD method (See Methods). Two distinct morphologies are known to be dominant in CVD MoS_2_ triangles^36^,^37^,^38^, where one involves a zigzag edge that comprises molybdenum and the other a zigzag edge that comprises sulphur. The former has a straighter edge than the latter and is entirely used in this work. An atomic force microscopy (AFM) image of the as-synthesized MoS_2_ sheet on a Si substrate shown in Fig. 1a indicates a smooth surface topography, combined cross-sectional and image histogram analyses of multiple topographic AFM images confirmed the MoS_2_ film thickness to be ∼0.75 nm as seen in Fig. 1a inset. Figure 1b shows a typical transmission electron microscopy (TEM) image of the synthesized MoS_2_ sheet, which reveals the periodic atom arrangement of the monolayer MoS_2_ film. The inset displays the corresponding diffraction pattern that indicates one set of hexagonal lattice structure, confirming that the synthesized MoS_2_ sheet has a monolayer structure with the highly crystalline quality. It is also noted from TEM-based energy-dispersive spectroscopy analysis (Supplementary Fig. 1) that the atomic percentage ratio between Mo and S is 1:2.

### Device fabrication

Triangular MoS_2_ monolayer films were then made into electronic devices by using the following procedures. Oxidized (300 nm) silicon substrates with millimetre-sized Au contact pads for electrical measurements were prepared by photolithography. In these standard chips, an 80 × 80 μm^2^ central region is prepared for electron beam lithography of nanodevice fabrication. Before transferring the as-synthesized MoS_2_ monolayer films onto the centre region, the sapphire-capped films were spin-coated with PMMA and immersed into 2 M NaOH solution to etch away the sapphire layer. The PMMA-capped MoS_2_ was first cleaned by deionized water and placed on the centre region. After removing PMMA, the monolayer MoS_2_ surface was further cleaned by chloroform. Tapping-mode AFM was used to determine the layer thickness and accurately identify the position of the individual MoS_2_ sheets for subsequent *e*-beam lithography. The selected MoS_2_ sheets were constantly measured by Raman spectroscopy to confirm the layer number, as shown in Supplementary Fig. 2. The measured energy difference between two Raman characteristic peaks at 384.3 and 405.2 cm^−1^ was 20.9 cm^−1^, indicating that the MoS_2_ film had a single-layer structure^44^, consistent with the AFM analysis in Fig. 1a. Nanoscale Au electrodes made by a standard *e*-beam lithographic technique were placed on top of the triangle MoS_2_ sheet, connecting it to the Au contact pads. To study the piezoelectric polarization direction, multiple contact electrodes around the triangle shape were intentionally designed. Figure 1c shows a typical device consisting of a triangular MoS_2_ monolayer device and several sets of source/drain (*S*–*D*) electrodes. The measurement setup for these MoS_2_ devices is shown schematically in Fig. 1d, in which the contact-mode AFM was used to apply a controlled mechanical load to the MoS_2_ monolayer.

### Mechanical–electronic coupling properties of the devices

**Figure 2:**
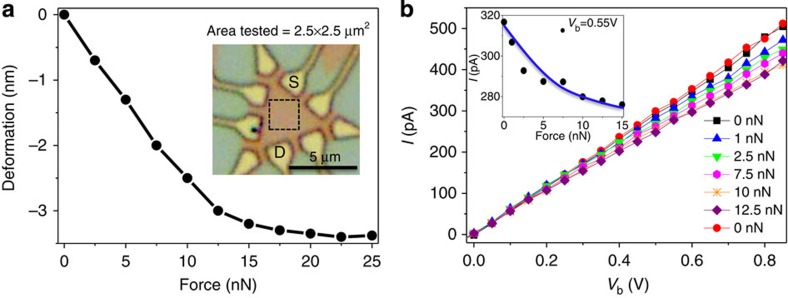
Deformation of the MoS_2_ monolayer and electromechanical properties of the device. (**a**) Deformation of the MoS_2_ monolayer under different forces applied by the AFM tip. Optical image of the measured MoS_2_ device (inset) denoting the tested 2.5 × 2.5-μm^2^ area by a black dashed rectangle. (**b**) *I–V*_b_ curves of the measured MoS_2_ device under a force applied in the centre of the tested region cycled from 0 to 12.5 nN and back to 0 nN, where the inset reveals the measured current under variable mechanical load at a fixed bias voltage of 0.55 V.

To identify the maximum deformation, which could be achieved in these MoS_2_ devices, the relationship between the applied force and deformation was measured through AFM-based force spectroscopy using the PeakForce Quantitative Nanomechanical Property Mapping mode. Deformation maps over a 2.5 × 2.5 μm^2^ area in the central region of MoS_2_ device shown in the inset of Fig. 2a were acquired under variable mechanical loading forces applied by AFM tip, as seen in Supplementary Fig. 3. Figure 2a provides the load-dependent deformation of the monolayer MoS_2_ device. Owing to physical constraints imposed by the underlying substrate, mechanical deformation of the MoS_2_ monolayer saturated when the applied force exceeded an average of 25 nN for all of measured devices. The current–voltage (*I–V*_b_) characteristics of these devices were investigated under variable mechanical loads using the circuit defined by a *S* and *D* as seen in the inset of Fig. 2a. By cycling the applied loading force from 0 to 12.5 nN and back to 0 nN with the AFM tip in contact with the centre of the denoted area, measured *I–V*_b_ curves shown in Fig. 2b reveal a decrease in current with increasing force and this decrease can be reversed when the strain was released. It can also be clearly seen that the measured current through the device at a fixed voltage (0.55 V) monotonically decreased as deformation increased, as shown in the inset of Fig. 2b.

**Figure 3:**
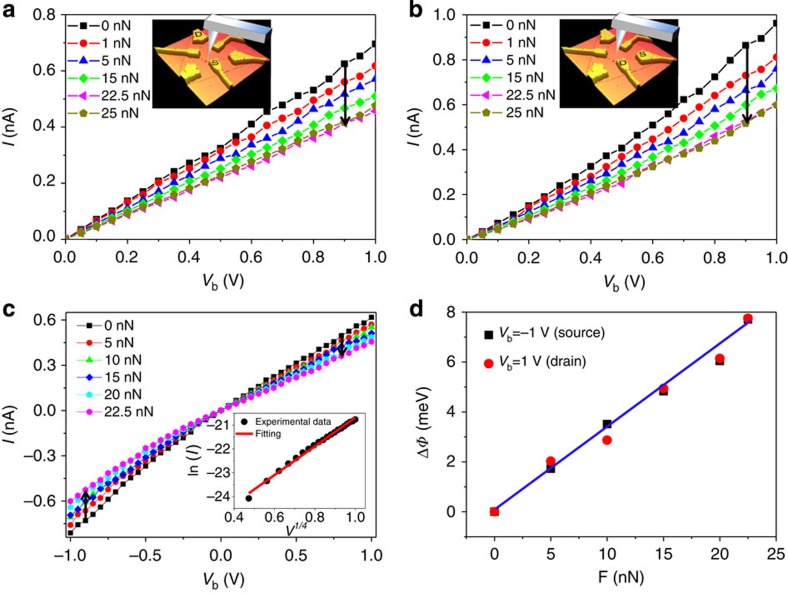
Electromechanical behaviour of a MoS_2_ device measured between different *S*–*D* electrodes. (**a**,**b**) Typical *I–V*_b_ characteristics of the device at different applied forces in the centre of the device measured between two *S*–*D* electrodes shown in the inset. (**c**) The *I–V*_b_ curves with both positive and negative bias voltage from −1 to 1 V. The inset is the fitting of ln*I* as a function of *V*^1/4^ by the *I–V*_b_ curve without strain using the thermionic emission–diffusion theory for a reversely biased Schottky barrier. The black dotted lines are experimental data points and the red line is a linear fitting. (**d**) The derived change of the barrier height as a function of applied force at a *D*–*S* bias of −1 and 1 V, respectively. The blue line is a linear fitting.

To further investigate the coupling effect of mechanical deformation and electric field, *I–V*_b_ curves similar to those shown in Fig. 3a,b were acquired in the central region of the sample under variable load, using different *S*–*D* electrode pairs as marked in the inset, respectively. It is noted that before the application of a mechanical loading force, different *S*–*D* combinations demonstrated small variations in their *I–V*_b_ characteristics with device conductance of the same order of magnitude. We attribute this to differing levels of electron scattering as mobile electrons traverse through different distances. Application of a loading force at the centre of the device results in a significant drop in the measured current that scales directly with the magnitude of the applied force. Figure 3c displays *I–V*_b_ curves in other two measured electrodes when a bipolar sweep voltage was applied to the device from −1 to 1 V. The same trend of the current change at the positive and negative bias was obtained. The observation of this phenomenon between all S–D combinations indicates that the position and directionality of the measured electrodes are not relevant to the deformation-induced modulation of conductance.

Along the electron transport path from the S to D electrodes, there is a back-to-back metal–semiconductor–metal contact. For carrier transport through a metal–semiconductor barrier, tunnelling effects dominate when the semiconductor is highly doped, whereas the thermionic emission dominates when the semiconductor is slightly or moderately doped. As the MoS_2_ employed in this work is intrinsic *n*-type and the measurements were carried out at room temperature, current through the reversely biased Schottky barrier *φ*_s_ can be given by a classic thermionic emission diffusion theory (for *V*>>3*kT/q*)^45^:


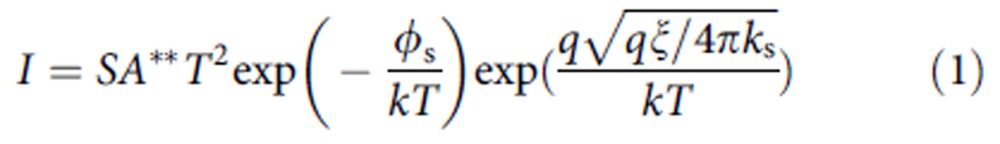



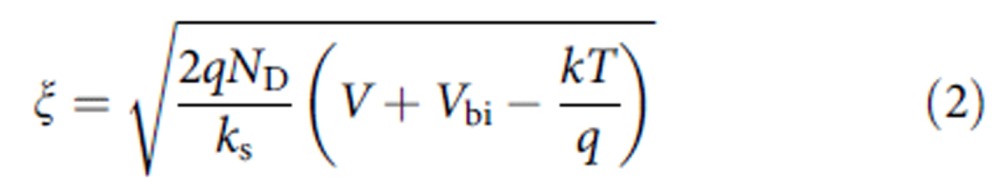


where *S* is the area of the contact junction, A** is the effective Richardson constant, *q* is the electronic charge, *k* is the Boltzmann constant, *T* is the temperature, *N*_D_ is the donor impurity density, *V*_bi_ is the build in potential at the barrier and *k*_s_ is the permittivity of MoS_2_. To evaluate whether this equation can precisely describe the observed phenomenon, ln*I* is plotted as a function of *V*_b_^1/4^ by using the data provided by the *I*–*V*_b_ curve without strain in the inset of Fig. 3c. The curve shows that the experimental data is fitted well with the linear model. This not only indicates that the thermionic emission–diffusion model is the dominant working process in the fabricated devices, but also shows that the theory can be applied to extract the Schottky barrier from experimental data. If *S*, A**, *T* and *N*_D_ are constant, the barrier height could be retrieved from the ln*I*–*V* plot^45^. Subsequently, the deformation-induced change of Schottky barrier height can be determined by


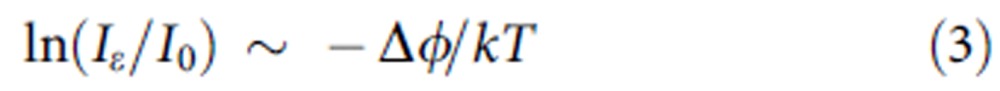


where *I*_ɛ_ and *I*_0_ are the currents measured through the MoS_2_ at a fixed bias with and without being deformed, respectively. The results are plotted in Fig. 3d for two biases of −1 and 1 V, indicating that the change of barrier height Δ*φ* on *S* and *D* contacts has an approximately linear relationship with applied forces and both the barrier heights at the *S* and *D* contacts were increased with increased deformation. Moreover, the Δ*φ* has the same trend at the other bias voltages, which is not very sensitive to the choice of bias voltage.

**Figure 4:**
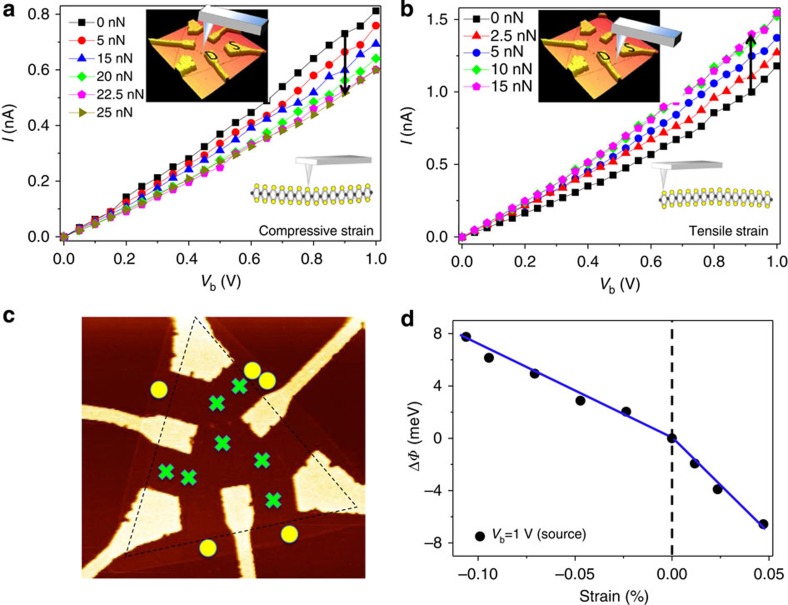
Electromechanical behaviour of a MoS_2_ device under compressive and tensile strain. *I–V*_b_ characteristics of the MoS_2_ device at different applied forces under compressive (**a**) and tensile (**b**) strain when applying forces at locations denoted in upper insets resulting in compressive/tensile strain as shown schematically in lower insets. (**c**) The relation of loading location to tensile/compressive strain, where experimental observations indicate that the MoS_2_ monolayer undergoes tensile strain when force is applied near the edges (yellow circles) versus compressive strain when applied at the centre (green crosses). (**d**) The derived change of the Schottky barrier height as a function of strain at a bias voltage of 1 V.

To better understand the origin of mechanical tuning of electronic properties in these devices, transport behaviours were compared by applying the mechanical force at distinct spatial locations on the MoS_2_ monolayer. Figure 4a,b provides the corresponding *I–V*_b_ curves measured at two fixed *S*–*D* electrodes, but with the AFM tip in contact at the centre and near the edge of the triangular film, respectively. The results reliably revealed a decrease in the measured current with increasing force applied at the centre (upper inset of Fig. 4a), but an increase in measured current with increasing force applied near the edge (upper inset of Fig. 4b) in all MoS_2_ monolayer devices. It was anticipated that both tensile and compressive strain result from localized deformation of the MoS_2_ monoloayer. The relation between spatially defined deformation and tensile/compressive strain was examined by positioning the AFM tip at various positions across the device as shown in Fig. 4c. The film can be imagined to be concave under compressive strain when a mechanical load is applied to the central region of the device (lower inset of Fig. 4a), whereas the surface is convex under tensile strain due to deformation near the edges (lower inset of Fig. 4b). These two different conditions of deformation position result in two bending cases of the film. According to experimental observations and our interpretation, tensile strain was observed by applying a mechanical load outside the dashed-line triangle region at points indicated by yellow circles in Fig. 4c. In contrast, compressive strain occurred when the contact region was inside the dashed-line triangle shape as indicated by green crosses. These observations strongly suggest that the film can be reliably switched between states of compressive and tensile strain.

The local strain *ɛ* of the monolayer MoS_2_ can be estimated as:


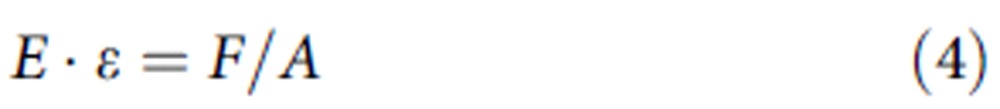


where *E* is Young's modulus, *F* is the applied force and *A* is the cross-sectional area of AFM tip. By approximating the nominal radius of the AFM tip (Bruker ScanAsyst-Air) to be 5 nm, the contact area *A* (*πr*^2^) is found to be roughly 78.5 nm^2^. *E* is known to be ∼270 GPa for monolayer MoS_2_ (ref. 14), thus the strain can be derived by the following equation:


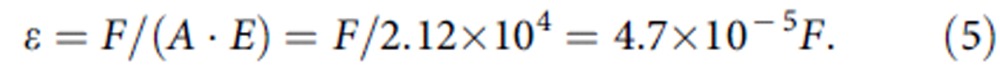


The relationship between the changes of barrier height Δ*φ* and the applied strain can therefore be obtained. Figure 4d shows the barrier height changes at bias voltage of 1 V as a function of strain, indicating that the change of barrier height increases with increasing compressive strain and decreases with increasing tensile strain. The asymmetry relation between compressive and tensile strain can be explained by differences in local strain-induced charge polarization. For the case of local tensile strain near the edge, the induced charge effectively accumulates on the zigzag edge. As compared with the local compressive strain at the centre, the induced charge is likely to be scattered by other electrons/charges during its migration to the zigzag edges. This may be caused by the fact that the barrier height change under tensile strain is different with that under compressive strain.

### Strain/force sensor

**Figure 5:**
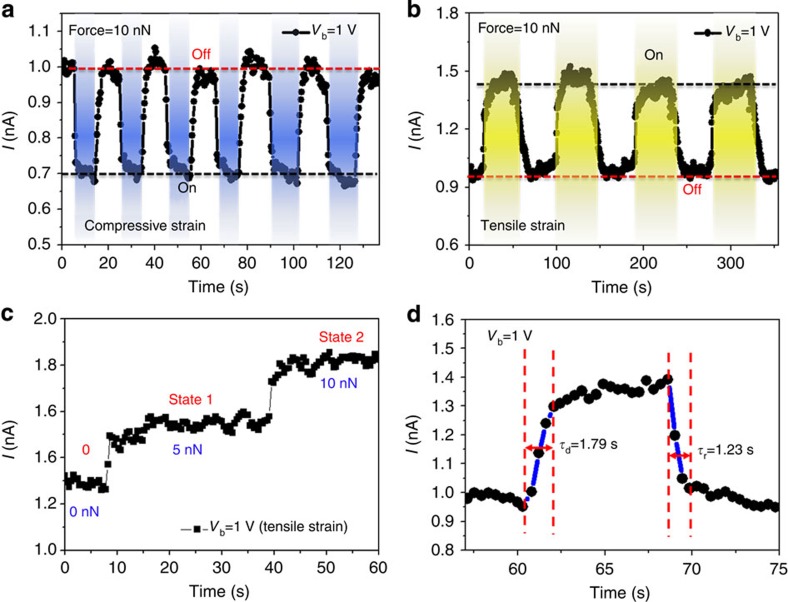
Current response of monolayer MoS_2_ strain sensor. Current response of CVD monolayer MoS_2_ device at repeated compressive (**a**) and tensile (**b**) strains at a fixed bias voltage of 1 V. (**c**) Measured current response when the device is free of strain (0 nN) and under mechanical loads equivalent to 5 and 10 nN, respectively. (**d**) Rise time and decay time of the MoS_2_ piezotronic device.

To investigate the observed device response towards sensor applications, the induced device current was measured as a function of time at a fixed bias voltage of 1 V under a periodically switched applied load. Figure 5a,b shows the time-resolved measurement of the device current response under compressive and tensile strain, controlled by the spatial location of the loading force, respectively. While applying a loading force of 10 nN, the measured current is immediately decreased under compressive strain and increased under tensile strain. The response was highly repeatable in many on/off cycles, indicating the stability of the device. The accessibility of multiple steady states in the device, characterized by the measured current under different loading forces (0, 5 and 10 nN) as shown in Fig. 5c, will enable the development of logic circuit applications based on these triangular MoS_2_ monolayer devices. The response time of the MoS_2_ strain sensor was evaluated as shown in Fig. 5d, in which the rise time and decay time were about 1.79 and 1.23 s, respectively. The performance of a strain sensor was also characterized using gauge factor, which is defined as [Δ*Ι*(*ɛ*)/*Ι*(0)]/Δ*ɛ*. The highest gauge factor in our CVD monolayer MoS_2_ strain sensors (Supplementary Fig. 4) was ∼1,160, a value much larger than that of the conventional metal sensors (1∼5), graphene (∼2)^46^ and doped-Si (∼200)^47^, and even greater than that of the highest reported gauge factor for CNTs (∼1,000)^48^. This increase can be attributed to the piezoelectric polarization and the detailed mechanism discussed in the next section. These results clearly demonstrate the utility of triangular MoS_2_ monolayer devices as force/strain sensors, to monitor the mechanical changes at the nanoscale range or smaller.

## Discussion

**Figure 6:**
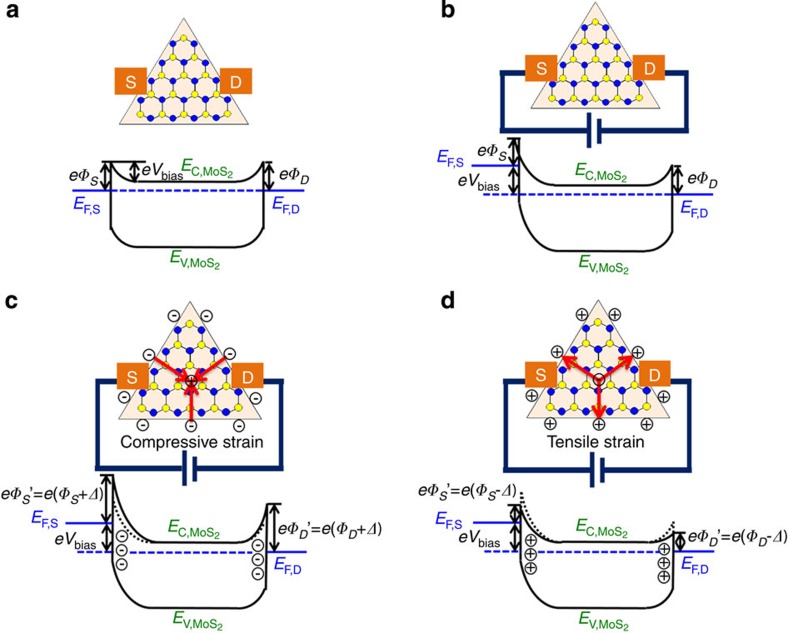
Band diagrams of the triangle monolayer MoS_2_ piezotronic device. (**a**) Energy band diagram of the device without bias voltage. Schottky barrier has similar barrier heights at the *S* and *D* contacts. (**b**) Energy band diagram of the device with an external bias. The quasi-Fermi level is raised at the source contact. (**c**) Negative polarization charges induced on three zigzag edges of MoS_2_ under a local isotropic compressive strain, depleting free electrons near the contact interface and increasing the SBHs at both contacts. The asymmetry of band diagram is the result of the bias. (**d**) Positive polarization charges induced on three zigzag edges of MoS_2_ under a local isotropic tensile strain, attracting free electrons near the contact interface and decreasing the SBHs at both contacts. The red arrows represent the directions of polarization. *E*_F_ is Fermi level of monolayer MoS_2_, E_C_ is conduction band, *E*_V_ is valence band, *V*_bias_ is the external bias and *Δ* is the piezopotential induced the change of barrier height.

It has been theoretically reported that due to lack of centrosymmetry in crystal structure, MoS_2_ with an odd number of layers under strain will give rise to in-plane piezoelectric polarization charges induced at the zigzag edges^24^. Here, an AFM tip has been used to apply controlled, local isotropic strain rather than uniaxial strain. In-plane charge polarization radiates circularly from the centre towards the zigzag edges in the case tensile strain, or in an opposite way under compressive strain, instead of along one direction as in the case of uniaxial strain^38^. In the case of a triangular monolayer MoS_2_ under local isotropic strain, the induced polarization charges are in the presence of three discrete zigzag edges. Band diagrams of triangular monolayer MoS_2_ piezotronic devices are provided in Fig. 6, to explain the underlying working mechanism. First, the presence of metal contacts with the semiconducting monolayer of MoS_2_ establishes the same Schottky barriers height (SBH) at both sides of contacts, as shown in Fig. 6a. When connected to an external power supply for electrical measurements, the quasi-Fermi level of the MoS_2_ device is raised at one of the contacts as seen in Fig. 6b. However, the barrier heights at the metal sides on both contacts remain the same. Next, placing the AFM tip in contact with the centre of the triangular MoS_2_ device produces local isotropic compressive strain in the monolayer and negative polarization charges are induced at three zigzag edges of the triangular MoS_2_, as shown in Fig. 6c. Finally, an induced negative piezopotential at the MoS_2_ side depletes free electrons near its interface with the metal, thereby increasing SBHs at both contacts and producing a decrease in the measured current under compressive strain. In contrast, positive polarization charges are induced at the three zigzag edges of the MoS_2_ triangle under a local isotropic tensile strain as shown in Fig. 6d. In the tensile condition, positive-induced polarization charges attract free electrons near the interface between the metals and the MoS_2_, resulting in decreased SBHs at both contacts and an increase in the measured current. The conductivity of the MoS_2_ device is clearly sensitive to changes in SBH and the measured current can be readily modulated by strain-induced charge polarization. It is noteworthy that the induced polarization charge with the same sign around three zigzag edges of the triangle MoS_2_ is expected to observe symmetrical modulation of carrier transport between any two contacts in this experiment. This was evidently verified in the observed *I–V*_b_ curves (shown in Fig. 3c). Based on the proposed mechanism, the piezoelectrically induced transport behaviour has been shown to be robust and reproducible. However, the piezoelectricity of nanomaterials may be affected by many factors, including the geometric size, crystal orientation, temperature, surface piezoelectricity and non-local effects. Although new electromechanical theories have been proposed in recent years^23^,^49^,^50^, great efforts are still required to understand the new physics of piezoelectricity in nanomaterials.

In summary, we have performed a thorough experimental study to confirm the piezoelectricity of CVD-grown triangular monolayers of MoS_2_ through the application of local isotropic deformation. The proposed working mechanism was confirmed to be the result of strain-induced in-plane charge polarization at the zigzag edges. These piezoelectric polarization charges alter Schottky barrier heights at both contacts and thus produce a change in conductivity of the MoS_2_ devices. A novel strain/force sensor using these MoS_2_ devices was also demonstrated. The highest gauge factor was found to be more than 1,000, a value much larger than that of the conventional metal sensors and comparable with that of the highest known gauge factor for CNTs (∼600–1,000). The merit of these triangular MoS_2_ devices lies in their suitability for the development of multi-directional nanoforce detectors. Experimental observations of the piezoelectric effect in 2D materials are expected to enable more applications in new electronic components such as touch sensors, energy generators and bio-integrated systems, as well as through their integration with silicon-based CMOS technology for achieving augmented functionalities.

## Methods

### Growth of atomic monolayer MoS_2_

High-quality monolayer MoS_2_ films were synthesized by CVD under the following conditions. Pure MoO_3_ powder was placed in a ceramic boat at the centre of furnace and cleaned sapphire or silicon substrate was placed faced down on the downstream side adjacent to the ceramic boat. A separate ceramic boat with sulfur powder was placed on the upstream side next to the MoO_3_ powder. The furnace was heated from room temperature to 650 **°**C at a rate of 25 **°**C min^−1^. After reacting for 3 min at 650 **°**C, the furnace was naturally cooled down to room temperature. In the optimized synthesis condition, monolayer MoS_2_ films with triangular shapes were produced over the entire substrate with a repeatability of ∼90%.

### TEM and Raman spectra measurement

The microstructures and morphologies of the nanostructures are characterized by a FEI Titan TEM. Raman spectrums were taken by a Horiba HR800 system with laser excitation wavelength of 532 nm.

### AFM and electrical characterization of devices

All AFM-based methods employed the Dimension Icon scanning probe microscope (Bruker Nano, Inc.) in ambient conditions using SiN probes (ScanAsyst-Air, calibrated spring constants of 0.3–0.5 N m^−1^ and nominal tip radius of 2∼5 nm). Cantilever spring constants were calibrated using the Sader method. Topographic characterization was carried out in the PeakForce Tapping mode. The PeakForce Quantitative Nanomechanics mode was used to characterize mechanical deformation of the MoS_2_ sample at variable mechanical loads. A combination of force versus distance and force versus separation spectra enabled accurate calibration of force-dependent deformation images acquired in the PeakForce Quantitative Nanomechanics mode. Contact mode was used for point deformation of the MoS_2_ monolayer devices under a fixed mechanical force throughout acquisition of respective *I–V*_b_ spectra. Electrical properties of fabricated devices were measured with a semiconductor parameter analyser (Keithley 4200) at room temperature.

## Additional information

**How to cite this article:** Qi, J. *et al*. Piezoelectric effect in chemical vapour deposition-grown atomic-monolayer triangular molybdenum disulfide piezotronics. *Nat. Commun*. 6:7430 doi: 10.1038/ncomms8430 (2015).

## Supplementary Material

Supplementary Information Supplementary Figures 1-4
