# Supplementary material for: Piezoelectric effect in chemical vapour deposition-grown atomic-monolayer triangular molybdenum disulfide piezotronics
Source: Nat Commun. 2015 Jun 25;6:7430. doi: 10.1038/ncomms8430 (PMC4491182; doi:10.1038/ncomms8430)
Supplement: Supplementary Information — Supplementary Figures 1-4 [file ncomms8430-s1.pdf]

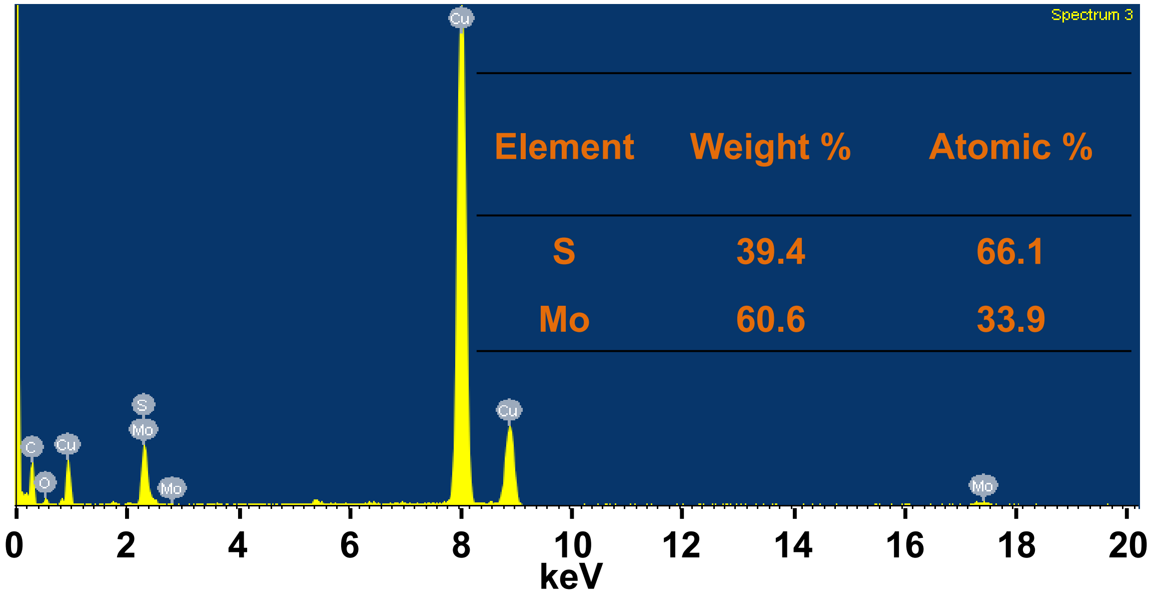

Supplementary Figure 1 TEM-based energy dispersive spectroscopy (TEM-EDS) analysis of the composition ratio of monolayer MoS_2 triangles.

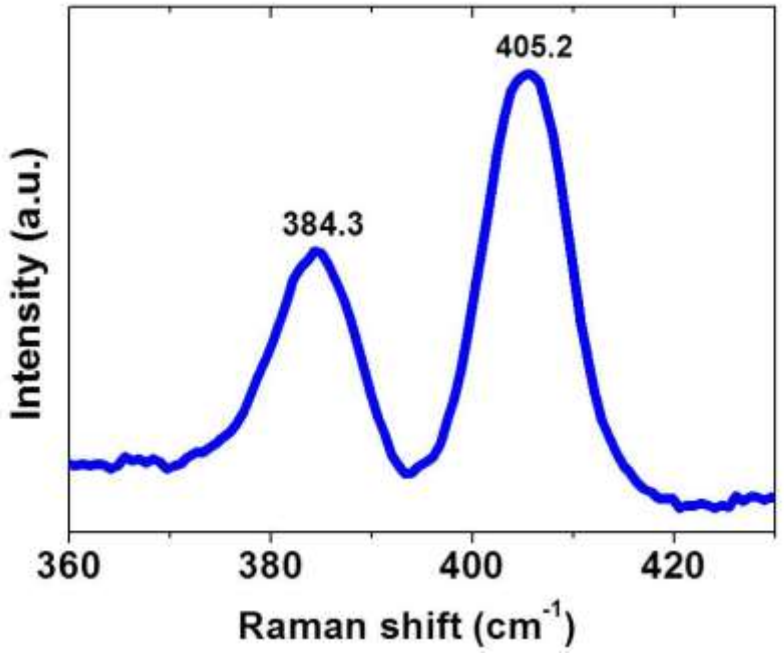

Supplementary Figure 2 Raman spectra for the transferred monolayer MoS_2 on silicon dioxide.

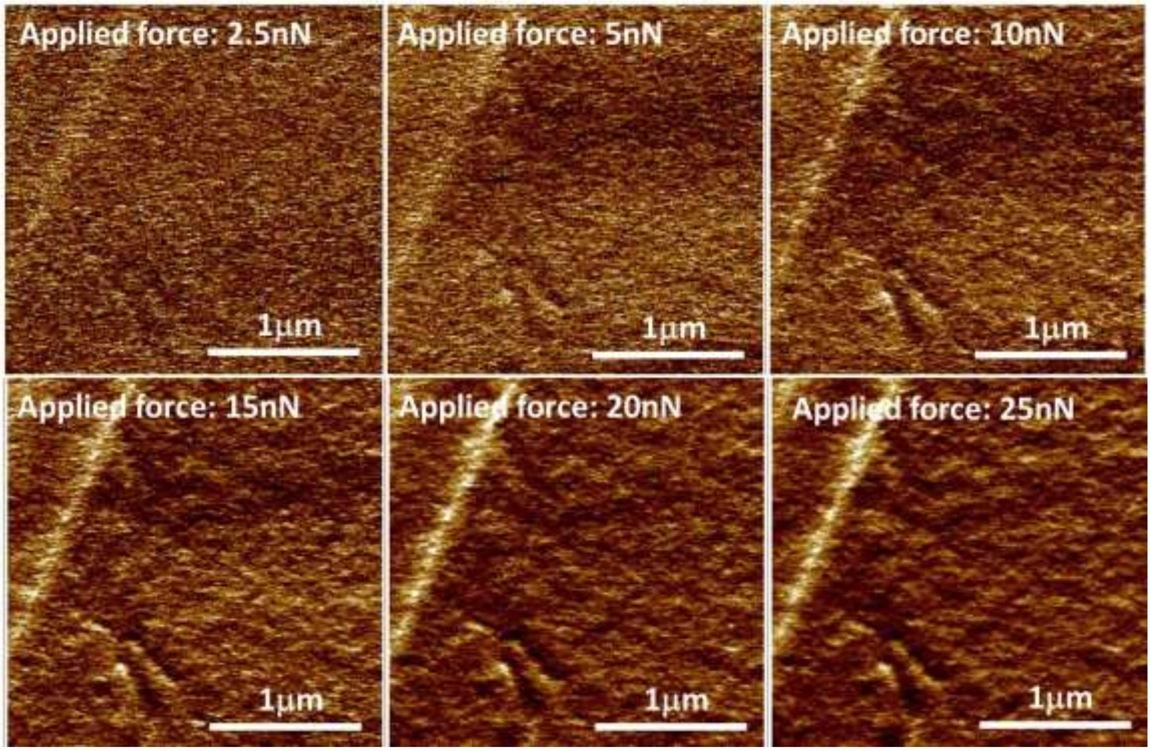

Supplementary Figure 3 Deformation images acquired over a $2.5\times2.5\mu m^2$ area in the central region of MoS_2 device under variable mechanical loading forces applied by AFM tip.

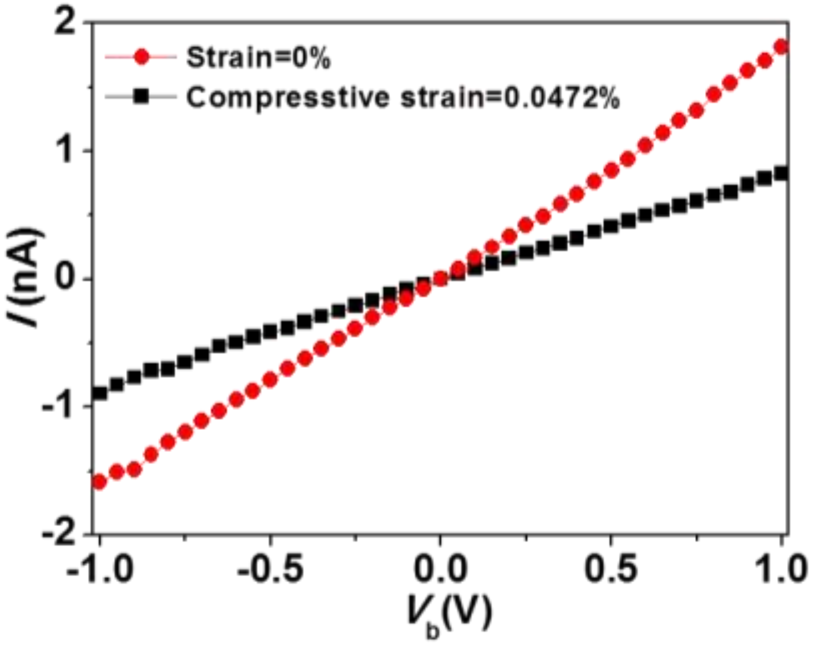

Supplementary Figure 4 I-V_b curves for the highest gauge factor of 1160 in our CVD monolayer MoS_2 strain sensor under free of strain and a mechanical load equivalent to 10nN (strain=0.0472%).
